# Vitamin A–Not for Your Eyes Only: Requirement for Heart Formation Begins Early in Embryogenesis

**DOI:** 10.3390/nu2050532

**Published:** 2010-05-25

**Authors:** Maija H. Zile

**Affiliations:** Department of Food Science and Human Nutrition, Michigan State University, East Lansing, MI 48824, USA; Email: zile@anr.msu.edu Tel.: +1 (517) 355-8474; ext. 127

**Keywords:** vitamin A-deficient, quail embryo, retinoic acid, gene regulation, heart morphogenesis

## Abstract

Vitamin A insufficiency has profound adverse effects on embryonic development. Major advances in understanding the role of vitamin A in vertebrate heart formation have been made since the discovery that the vitamin A active form, all-*trans*-retinoic acid, regulates many genes, including developmental genes. Among the experimental models used, the vitamin A-deficient avian embryo has been an important tool to study the function of vitamin A during early heart formation. A cluster of retinoic acid-regulated developmental genes have been identified that participate in building the heart. In the absence of retinoic acid the embryonic heart develops abnormally leading to embryolethality.

## Abbreviations

RA: all-*trans-*retinoic acid
VAD: vitamin A-deficient
HH: Hamburger & Hamilton developmental stages for avian embryo
ss: somite stage
IFT: inflow tract

## 1. Function of Vitamin A, Retinoic Acid, Retinoid Receptors and Gene Regulation

It has been more that 80 years since the discovery of vitamin A in 1913 [1,2] and much has been learned about this important vitamin [3,4,5,6,7,8,9,10], but still much more remains to be done to fully understand the complex functions of this micronutrient. The following minireview provides a brief overview of the major questions that have been answered regarding vertebrate embryonic development, with a focus on heart formation. 

The need for proper vitamin A nutrition throughout the life cycle is well established and involves the requirement for this vitamin in vision, reproduction and growth, but the molecular mechanism(s) of action of this micronutrient are still under investigation. Except for its role in vision, the functions of vitamin A are mediated by its physiologically active form, all-*trans*-retinoic acid (RA). The pleiotropic effects of vitamin A are attributable to a multitude of RA-linked transcriptional pathways involved in a number of cellular growth and differentiation processes [4,7,8,11]. RA functions as the ligand for specific nuclear transcription factors, the RA receptors RARα, β and γ [12,13,14,15,16] and PPARβ/*, the orphan peroxisome proliferator-activated receptor β/δ [17,18,19]. The RARs form heterodimers with the retinoid X receptors, RXRα, β and γ, and the heterodimers bind to specific RA-responsive elements in the target genes; their transcriptional effects (activation or repression) depend on the recruitment of coactivators and corepressors, respectively, in the presence or absence of retinoic acid [11,20]. The RARs and RXRs are known as the retinoid receptors. In addition to the transcriptional actions, RA is also involved in rapid nongenomic actions [13,21,22]. RA regulates more than 500 genes and is among the most important signaling molecules in vertebrate ontogenesis.

## 2. Vitamin A in Embryonic Development

The requirement of vitamin A for normal embryonic and fetal development is known from many nutritional studies [2]. It was recognized already in the 1930ies that maternal insufficiency of vitamin A during pregnancy results in fetal death or abnormalities in the offspring that include abnormal heart and central nervous system development [23,24,25]. While it is known that vertebrate embryos have the machinery for vitamin A metabolism and for the generation of all-*trans-*retinoic acid [26,27,28], the underlying cellular and molecular events of early embryonic development that are regulated by endogenous vitamin A-active molecules have not been fully elucidated. 

The origin and evolution of RA signaling during embryonic development has been traced as far back as the metazoans [29,30]. Both vitamin A deficiency and excess have profound adverse consequences on morphogenesis and organogenesis in the vertebrate embryo [9,31,32,33,34,35,36,37,38,39,40], implying that normally there is a tight regulation of the endogenous vitamin A-active form, all-*trans*-RA. Exogenously applied vitamin A-active compounds have been used to manipulate and perturb normal embryonic development, and have provided evidence that almost every organ or tissue system can be severely affected by RA if the embryo is treated with it at a critical time in development [39,40,41,42,43,44]. These studies have revealed that RA via its nuclear receptors can also regulate the expression of developmental genes. However, in these approaches concentrations well above endogenous levels were used and thus may not reflect the physiological functions of vitamin A in normal development.

A large amount of valuable information regarding the function of vitamin A during embryonic and fetal development has been gathered by the use of transgenic mice with mutations or knock-outs of the retinoid receptor genes [7,11,45,46] as during development there is a widespread and complex expression of these receptors [14]. Many of the abnormalities in these mutant mice resemble those observed in fetuses from the vitamin A-deprived animals reported earlier. Among other important approaches in the studies of molecular mechanisms of retinoid action in development is the use of *in vivo* mouse embryo model systems in which the function of vitamin A has been diminished by interfering with vitamin A metabolism. These approaches have added important information about RA function and signaling pathways in embryos and include knock-outs (KOs) of the genes for RA synthesizing enzymes Raldh2 [47,48], Raldh1 [49] and Raldh3 [50,51], the KOs of the RA degrading enzyme Cyp26 [52,53,54], double KO of Raldh2/Cyp26 [55], the combination KO of Cyp26b1/ RARγ [56] and the zebrafish Cyp26b1 mutant [57]. Another approach is to use nutritional deprivation of vitamin A in a rat model to obtain near-vitamin A deficiency in the dams and to target embryonal vitamin A insufficiency to distinct gestational windows. These rat embryos exhibit specific cardiac, limb, ocular, lung and central nervous system abnormalities, some of which have certain features similar to those reported in retinoid receptor knockout mice [9,35,58,59,60,61].

Altogether, while numerous studies have addressed the function of RA in embryonic development, the models used have resulted in diverse phenotypes because of an incomplete inactivation of RA signaling [62,63,64] or in the case of retinoid receptor knockouts, they do not provide a complete and clear vitamin A deficiency phenotype due to retinoid receptor redundancy [64] and the broad role of the RXRs as co-receptors for other nuclear receptors in non-retinoid signaling pathways [7,11]. Recent comprehensive reviews on the genetic dissection of retinoid signaling pathways point out the shortcomings of germline mutagenesis in addressing the physiological functions of vitamin A [15,65]. The absolute essentiality of vitamin A for early embryogenesis is unambiguously demonstrated in the vitamin A-deficient (VAD) avian embryo, an ideal model to study the function of vitamin A in a vertebrate embryo during early development. These completely VAD embryos develop gross abnormalities in the cardiovascular and central nervous systems and trunk and die early in embryonic life [31,32,66,67,68,69]. Significantly, the VAD avian embryo can be rescued and normal development restored by the administration of the physiological ligand for RARs, all-*trans*-retinoic acid, or its precursor, retinol, during a critical, RA-requiring time window in which important developmental events are specified.

## 3. Vitamin A Deficiency, Heart Development and the Avian Embryo

Maternal insufficiency of vitamin A during pregnancy results in fetal death or abnormalities in the offspring including abnormal heart development [23,24,25]. While it is generally impossible to link an early miscarriage or a fetal death to a specific maternal nutritional status, it is important to recognize the extreme sensitivity of the very early embryonic genome to epigenetic influences, including those of maternal environment. This is particularly relevant when discussing cardiovascular development since pediatric cardiovascular abnormalities account for 8% of all deaths during the first year of life, and congenital heart defects in the Western world are as high as 12/1000 live births, representing the majority of all congenital malformations [70,71,72,73]. The etiology of these malformations is largely unknown, but inadequate or inappropriate nutritional cues during early heart morphogenesis are likely contributors to these birth defects. This has been clearly evidenced in cases of insufficient folate supplementation during pregnancy which leads to neural tube defects in newborn. While a genetic predisposition to congenital heart disease is likely present in certain cases, it is also likely that another insult such as vitamin A insufficiency or a disturbance in vitamin A metabolism or function during embryogenesis, contributes to the cardiovascular defects observed clinically.

Vitamin A deficiency is not prevalent in the U.S. but marginal insufficiency or disturbances in vitamin A metabolism such as may be caused by environmental pollutants or alcohol abuse during pregnancy, are important risk factors. It is, however very difficult to demonstrate the link of vitamin A insufficiency in the mother to congenital abnormalities in the offspring, as vitamin A requirement begins very early during embryogenesis and involves many genes that critically impact early development. Presently there is no understanding of the molecular basis of cardiovascular birth defects. The critical vitamin A-requiring developmental events that take place in the early avian embryo during heart formation [67] coincide with the first 2-3 weeks of human pregnancy and may be severely compromised if maternal vitamin A intake is marginal or if there is interference with vitamin A function during pregnancy. The high incidence of vitamin A deficiency in developing countries may account for the increased incidence of heart malformations in these populations [74]. The WHO reports that millions of pregnancies yearly are carried by women with insufficient vitamin A nutrition [75].

The cardiovascular system is the first physiologically functioning organ system to form in the embryo. The development of the embryo is critically dependent on a normal and functional development of this system so as to obtain nutrients and growth factors. While it has been well documented that vitamin A-active compounds have an important role in heart development [32,34,76,77,78], the underlying morphological, cellular and molecular events of early embryonic development that are regulated by endogenous vitamin A-active molecules have not been elucidated. In order to prevent or ameliorate cardiovascular birth defects that may be caused by disturbances in vitamin A function or insufficiency during early development, it is imperative to understand how vitamin A works during the formation of the heart and the vascular system. An excellent recent review offers the most up-to-date information on the role of vitamin A in mammalian heart development, and describes the major discoveries at the gene level that have been made with the use of rodent models at various selected stages of heart morphogenesis [78]. 

The purpose of the present minireview is to point out the advances that have been made regarding the role of vitamin A in the very early events of vertebrate vasculogenesis and heart formation, not addressed in the mammalian models. This research has been possible with the use of a novel vitamin A-deficient (VAD) avian (quail) embryo model in which one can examine biological actions at cellular and molecular levels attributable solely to vitamin A. Our laboratory has utilized this model for the *in vivo* examination of the physiological function of vitamin A in vertebrate embryogenesis with a focus on early cardiovascular development; the following advances have been made: a). The confirmation of the absolute dependency on the presence of vitamin A for the morphogenesis of the cardiovascular system and for avian embryo survival [79] that had been observed earlier [3,80]. The hallmark of vitamin A deficiency in the avian embryo is a grossly abnormal cardiovascular system, characterized by an absence of vascular networks and by a ballooned, non-compartmentalized, randomly-positioned heart, without an inflow tract at the posterior site of the heart [79]; these embryos die by embryonic day four; b). The discovery of a critical time window when the presence of RA is absolutely essential for normal embryogenesis to proceed; this time coincides with the initiation of heart morphogenesis and the organization of endothelial cells into vascular networks [67] and corresponds to the first 2–3 weeks of human pregnancy. If RA is administered to the VAD quail embryo during this critical time or prior to it, the embryo is rescued and develops normally. It is important to note that severe cardiac malformations are observed in an estimated 10% of early miscarriages [73]; some of them may be linked to disturbances in vitamin A function during the early critical time of heart formation; c). The identification of a cluster of developmental genes that are regulated by RA during early heart formation; they include the cardiac transcription factor GATA4 [68], the heart asymmetry genes *Nodal, Snail* and *Pitx2* [66], the global growth factor Bmp2 [81], the adhesion molecule N-cadherin [82] and the global growth regulator TGFβ2 [83]; and d). The discovery that all retinoid receptors are expressed in the early avian embryo (67, 84-86), but that during the critical RA-requiring developmental window RARα2, RARγ and RXRα are the active transcription factors transducing the RA signal to target genes [84,85]. 

## 4. Vitamin A is Required to Build the Link Between the Primordial Heart and its Blood Supply

Building of the heart is one of the many complex morphogenetic events during embryogenesis; it is coordinated by numerous growth regulatory molecules, including RA. In the VAD avian embryo, the predominant feature of the VAD phenotype is a failure of cardiovascular development, with embryo lethality linked directly to the absence of the cardiac inflow tracts, *i.e.,* these embryos lack the *sino atrial* tissue and their hearts are closed posteriorly, thus there is no blood supply to the embryo [32,79,80]. The administration of RA or retinol during or prior to the critical developmental time window when RA requirement begins in the early avian embryo (*i.e.,* the HH8 4/5 somite stage) rescues the VAD embryo, and it now develops with a completely normal phenotype [66,67,68].

The absence of a connection of the extraembryonal blood supply to the forming heart in the developing VAD heart pointed to a critical role for vitamin A in building the posterior heart and led to the hypothesis that the cardiovascular abnormalities associated with the VAD embryonic phenotype and the inevitable embryolethality are initially linked to an inability to form the heart inflow tracts (IFTs) in the absence of vitamin A. The building of the IFTs is a crucial event in early cardiogenesis and cardiovascular development. Vertebrate heart morphogenesis begins in the posterior heart forming region and proceeds anteriorly. The formation of the connection between the primordial heart and the extraembryonal vasculature is the first event that takes place in heart development; it occurs at the site of the formation of the future heart IFTs in the posterior region of the primordial heart. It is this event that lays the structural foundation for a subsequent normal heart morphogenesis. The devastating impact of the failure to form this linkage is clearly evidenced by the embryolethality of the VAD quail embryo [31,32].

Building of the heart IFTs involves cells from different lineages [87,88], and also includes the formation of the incoming blood vessels [89], which converge into vitelline veins at the posterior regions of the developing heart. The studies in our laboratory have been directed to more fully elucidate the embryonic molecular environment during early development that is required for the successful building of the heart IFTs. Presently there is a gap in the general knowledge regarding the formation of the cardiac IFTs, while there is an abundance of data describing the events subsequent to posterior heart formation [78]. Although the potential role of RA in this process has been discussed [90,91] the models used did not allow for an evaluation of the physiological role of RA at the very early stages of heart morphogenesis; this has been possible with the use of the completely VAD vertebrate embryo, the avian embryo. 

It is very difficult to clinically link vitamin A function to the important early event of cardiac IFT formation, since no specific birth defects or cardiovascular malfunctions in neonates or adults have been attributed to a faulty formation of the heart IFTs. However, it is of great importance to recognize, as our work suggests, that it is very likely that if severe malformations had occurred during early heart morphogenesis at the sites of the developing IFTs, they would have led to embryo lethality, thus no information about the specific cause of the death of such embryo would be available. Supporting this possibility are the observations that severe cardiac malformations are associated with an estimated 10% of early miscarriages [73]; some of them may be linked to disturbances in vitamin A function during the early critical time in heart formation.

Evidence for the importance of vitamin A in building the heart IFTs is based on the following discoveries in our laboratory: a) In the VAD embryo the cardiac IFTs do not form [31,32,79]; b) The cardiogenic transcription factor GATA4, normally expressed in the early developing heart, is severely diminished in the VAD embryo in the posterior heart forming regions at the sites of the future IFTs [68,81]. However, cardiomyocyte differentiation is not altered and the primitive heart tube forms in these VAD embryos, thus providing evidence that RA signaling is not required for these early heart morphogenesis events [68]; c) Additional support for the role of vitamin A in IFT building was obtained from the localization of Raldh2, the main RA synthesizing enzyme, in the posterior heart region during quail embryogenesis [85] and from the demonstration of expression of all retinoid receptors in the heart forming regions of quail embryos [67,84,85,86]; and d) A systematic examination of the early development in the VAD quail embryos revealed that in these embryos one of the earliest observable morphological defects is an abnormal formation of the heart IFTs which gradually become more narrow and finally close up, thus resulting in the absence of a connection between the developing heart and the extraembryonal blood supply to the heart [82].

## 5. Molecules Involved in Retinoic Acid-Regulated Building of the Heart Inflow Tracts

Many signaling pathways participate in the important and complex interactions to form a heart. The successive activation of different sets of genes for heart morphogenesis is by specific transcription factors and include Nkx2.5, Hox, Msx1 & 2 and GATA4/5/6 [92,93]. Since we already knew that the critical RA requirement is at the HH8 4/5 somite stage during neurulation and since this is also the time window when embryonic cell terminal differentiation is determined, our studies have focused on cellular and molecular events taking place at this developmental time and during the immediate events just prior to and just subsequent to it, but before heart chamber segmentation and formation of blood flow, so as to avoid secondary effects.

We have identified the following cluster of molecules that are regulated by RA during IFT formation: a) The retinoid receptors, which are also transcription factors, are expressed in the heart forming areas of the early quail embryo [84], but in the VAD embryos the expression of the RA receptor RARα2 is severely decreased in these regions [84]; blocking the expression of this receptor in normal embryos interferes specifically with the formation of IFTs [85] providing evidence for a specific role of RARα2 in regulating heart IFT morphogenesis; b) The cardiogenic transcription factor GATA4 may be a contributor to RA-regulated IFT formation, as normally GATA4 transcripts are particularly high in the posterior heart region [94], but in the VAD quail embryos they are severely diminished at these sites [68]; and c).The overexpression of N-cadherin [82] and TGFβ2 [83] observed in VAD quail embryos, is linked to the abnormal heart IFT formation; this is discussed in the following sections. 

From the above evidence it becomes clear that it is for the morphogenesis of the heart posterior region that the initial RA signaling input at molecular level is required for a subsequent normal cardiac morphogenesis and a normal embryonic development to take place, and that the abnormal cardiovascular phenotype associated with vitamin A deficiency in the avian embryo is the consequence of an initially disrupted program for posterior heart morphogenesis.

## 6. Retinoic Acid Regulates N-cadherin During Early Heart Formation [82]

Our recent work on the function of RA in heart IFT formation addresses the cell adhesion molecule N-cadherin (N-cad). Cell-cell and extracellular matrix interactions profoundly influence many signaling events, including cell migration, mitogenesis, apoptosis and differentiation, all of these events required for the morphogenesis of the heart. Thus we next examined the interrelationship between RA and N-cad, since this molecule is crucial for heart formation [88,95,96,97,98,99], including establishing cardiac left-right asymmetry [100], which is also regulated by RA [66]. The endocardial and mesodermal cells in the posterior parts of the cardiac crescent participate in the remodeling of the posterior heart and involve N-cad mediated cellular organization [88,99]. Vitamin A *via* RA has pleiotropic functions and thus is likely to participate in any one of these processes. These considerations led to the studies with N-cad and the discovery that RA regulates N-cad [82]. N-cad mRNA and protein were found to be expressed globally in the presomite through HH14 normal and VAD quail embryos, but the expression in VAD embryos in the early stages prior to HH10 was significantly higher than that in normal embryos, indicating that RA negatively regulates N-cad at these developmental stages. Functional analyses of the N-cad overproducing VAD embryos revealed that N-cad is involved in several RA-regulated cardiovascular events, *i.e.,* IFT morphogenesis, vascularization and cardiac looping, but not in the regulation of heart asymmetry. While the mechanisms remain to be elucidated, it is possible that too much N-cad disturbs mesodermal cell organization, since an excess of N-cad can interfere with cell movement and limit migration [95].

In order to obtain clues about the mechanism of the RA and N-cad relationship, we examined the time course of N-cad gene repression by the administration of physiological amounts of RA to VAD embryos. These studies revealed that RA does not exert a direct effect on the N-cad gene, as approximately 2 h elapsed before the *in ovo* administration of RA to the VAD embryo caused a normalization of the overexpressed N-cad gene. About 30–45 min are required for RA to induce transcription in the VAD embryo of a direct RA target gene (with RARE), such as RARα2 [84]. If RARα2 were the receptor involved in regulating N-cad expression, an altered expression of N-cad should have been seen in less than 2 hours. These observations suggest that RA exerts its effect on N-cad by an indirect mechanism, *i.e.,* that downstream of RA there exists a signaling pathway which, upon the activation by RA, regulates N-cad.

In further studies we obtained evidence that N-cad expression may be mediated by Msx1. The homeobox transcription factor Msx1 is known to be involved in heart morphogenesis [101,102]. Earlier we had observed that this gene is ectopically expressed in the heart forming regions of the VAD quail embryo [103]. Since Msx1 and N-cad both were found to be overexpressed in the VAD embryo during early heart morphogenesis and since N-cad mediated cell adhesion and cell sorting is known to be regulated by Msx1 [104], it was of great interest to determine if the excessive expression of N-cad in the VAD quail embryo is mediated by Msx1. The approach was to block the ectopic Msx1 expression in VAD embryos with antisense oligonucleotides specific to Msx1 and then assess N-cad gene expression. When the ectopically expressed Msx1 *g*ene was blocked in the VAD embryo, this also resulted in blocking the excessive expression of N-cad. From these findings we proposed that RA normalizes N-cad overexpression in VAD embryos by an indirect pathway that may be mediated by Msx1. Msx1 is sensitive to the regulation by RA, as the promoter in Msx1 has been found to have an enhancer responsible for RA induction [105]; there is also a possible binding site for RAR/RXR [106], as well as several transcription factor binding sites for RAR, RXR and the RAR/RXR heterodimers [107], but the structure of a RARE in this promoter has not been fully characterized. Additionally, the Msx1 promoter also has AP-1 sites [106,107], where RA/RAR can block Msx1 expression. RA is a well-known negative regulator of AP-1 responsive genes [108] and a potent inhibitor of AP-1 mediated growth signals [6,11]; suggested mechanisms include RA/RAR competing for coactivator proteins [109], RA/RAR disrupting cjun/cfos dimerization and RA/RAR inhibiting cjun phosphorylation [110]. These potential mechanisms could explain how the absence of RA results in Msx1 overexpression in the VAD embryo. Since Msx genes have been linked to avian cardiac development [111] and to the regulation of N-cad [104], the interrelationship between RA, Msx1 and N-cad warrants further examination.

The Msx genes are multifunctional patterning genes, expressed at sites where apoptosis occurs [112] and thus are strongly implicated as transcriptional regulators of apoptosis during development [113]. Apoptosis is a normal and prominent factor in ridding the embryo of cells which have fulfilled their function in morphogenesis; this also takes place during heart development [114]. The altered morphogenesis of the posterior heart region as well as the sparse vascular networks characteristic of the VAD embryo [82,89,115] may involve an increased apoptosis, since Msx1, an apoptosis gene, is upregulated in the VAD embryo [82,103]. This idea is supported by the observations of increased apoptosis in the VAD embryo in the central nervous system, the somites [116,117] and in the foregut [118]. Thus it is possible that Msx1 is affecting two different downstream signaling pathways, *i.e.,* one that regulates N-cad and another that is involved in apoptosis, both participating in the RA-regulated morphogenesis of cardiac inflow tracts. 

A very important observation in these studies is that in the early avian embryo endogenous RA is a negative physiological regulator of both Msx1 and, indirectly, of N-cad. We hypothesize that a critical endogenous level of N-cad is needed for normal early cardiovascular morphogenesis to occur and that this level is ensured by a stage-specific, developmentally regulated RA signaling. While such negative regulation by RA is the first observation regarding the *N-cadherin* gene, the potential for RA to be either a positive or a negative regulator is well known from *in vitro* studies and from observations that the transcriptional effects (activation or repression) of the retinoid receptors depend on the recruitment of coactivators and corepressors, respectively, in the presence or absence of RA [11,20]. Our studies have now confirmed such negative regulation also in the *in vivo* vertebrate embryo but the mechanisms remain to be elucidated. 

## 7. TGFβ2 is Negatively Regulated by Endogenous Retinoic Acid during Early Heart Formation

Our most recent studies have focused on TGFβ2 as a major regulatory target of RA during early embryonic development [83]. It is well known that TGFβs and RA share global growth regulatory characteristics, e.g., modulation of cell proliferation, apoptosis and differentiation [4,6]. Our observations that the entire VAD quail embryo phenotype can be rescued by the administration of RA during the vitamin A-sensitive developmental window [32,67,68], suggested that RA-regulated early cardiovascular morphogenesis is implemented by global growth factors such as TGFβs, as they regulate many developmental pathways. Futhermore, it is also well known that in many cells RA regulates TGFβ signaling [4,119,120,121,122,123], with TGFβ2 being the most sensitive isoform to tissue retinoid levels [124]. The above rationale led to testing the idea that RA regulates TGFβ2 signaling during *in vivo* embryonic heart development. The role of TGFβ2 in heart development at the time of initiation of heart morphogenesis had not been investigated in an *in vivo* vertebrate embryo nor in the context of an interaction with physiological levels of RA. In our work with TGFβ2 [83] we made the following important and novel observations: a) Endogenous RA is a critical negative *in vivo* regulator of TGFβ2 during early avian embryogenesis, evidenced by the overexpression of TGFβ2 in the VAD embryo. We hypothesize that the negative regulation of TGFβ2 by RA is via the AP-1 site in the TGFβ2 promoter [125]; b) RA regulation of TGFβ2 during early heart morphogenesis is at the level of transcription; for this RA-regulated activity TGFβ2 utilizes a Smad-independent pathway; and c) The use of the VAD avian embryo model revealed a major role of TGFβ2 in posterior heart development. We propose that during the early physiological events of vertebrate cardiovascular development a strictly regulated TGFβ2 activity by endogenous RA is essential for the initial heart IFT morphogenesis to take place involving the link-up of the primitive heart tube to the extraembryonal vasculature. These events are followed by the positioning of the heart tube to the right side and its subsequent looping. The events are separate and independent, and likely involve other developmental signals. Clearly, TGFβ2 is an important participant in the complex network of factors required for early heart morphogenesis. Some cardiovascular pathologies that are associated with elevated levels of TGFβ2 [126,127] may be linked to disturbed RA function during pregnancy. 

## 8. Summary and Conclusions

Many important questions about vitamin A have been answered since its discovery, and much has been learned about the effects of maternal vitamin A deficiency on fetal heart development [33,128]. Studies with rodent models [9,45,46,47,48,49,50,51,52,53,54,55,56,57,58,63,76,78,128,129,130,131,132,133] have significantly added to our understanding of the role of vitamin A in vertebrate heart formation, particularly during the later stages of heart morphogenesis, after the initial formation of the posterior heart when the heart inflow tracts link up with the extraembryonal vasculature. The studies with the avian embryo model, reviewed here, have extended the existing knowledge of vertebrate heart morphogenesis by providing insights into the essential role of vitamin A for the very early events in this complex process. 

**Figure 1:**
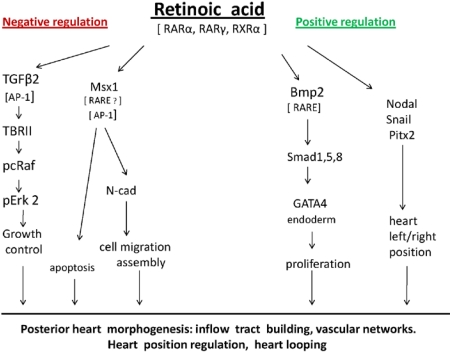
Potential retinoic acid signaling pathways involved in building the early embryonic heart. Retinoic acid (RA) is an embryonic morphogen, participating in heart morphogenesis. The effects of RA are mainly mediated by RA as the ligand for the transcription factors RARs. TGFβ2 [125] and Msx-1 [106,107] both have AP-1 sites in their promoters, that are negatively regulated by RA/RAR blocking AP-1 activity [6,11,125]. Potential binding sites for RAR/RXR have been identified in the Msx1 promoter [105,106,107]. Genes containing the RA responsive element, RARE, such as Bmp2 [137], are direct RA target genes, regulated by RA/RAR/RXR heterodimers. The potential signaling pathways are based on research data obtained in M. Zile’s laboratory [83].

Clearly, the complexity of cardiovascular development mandates that we analyze multiple gene pathways to evaluate the role of vitamin A and to lay the groundwork for subsequent mechanistic studies. Using the avian model we have identified a cluster of downstream effectors of the RA signaling pathways involved in vertebrate early cardiovascular morphogenesis. They include the heart asymmetry genes *Nodal,* *Snail* and *Pitx2*, the cardiogenesis transcription factor GATA4, linked to Bmp2. The retinoid receptor RARα2 is specifically linked to heart inflow tract formation, while RARγ is linked to heart asymmetry and looping. The cell-cell adhesion molecule N-cadherin is involved in several aspects of heart morphogenesis, including inflow tract formation, heart looping and vascularization, and in these events may be regulated by Msx1. TGFβ2 was found to be a major contributor to RA-regulated posterior heart morphogenesis. Importantly, RA was identified as a negative physiological regulator of the global growth factors N-cadherin and TGFβ2 during early heart formation. Potential RA signaling pathways involved in building the early embryonic heart are illustrated in Figure 1.

The elucidation of the pleiotropic functions of RA during embryonic development remains an active field of investigation. We are continuing the characterization of vitamin A-regulated genes involved in the formation of the early heart and the vascular system. Since early vertebrate embryonic heart development is similar across the species, including the human, many analogies can be drawn from such studies. Although much more work is needed to completely elucidate the role of vitamin A in heart formation, the presently available data provide strong, evidence-based information emphasizing the importance of an adequate and functional vitamin A environment during early pregnancy.

Understanding the molecular role of vitamin A in early embryonic and fetal events is imperative in developing intervention strategies during human *in utero* development so as to decrease the incidence of cardiovascular birth defects and thus minimize problems that are likely to arise during adult life from an unfavorable *in utero* environment. The exploration of origins of disease and various pathologies as linked to the environment of vitamin A and other micronutrients during critical early stages of embryonic development is only recently gaining a momentum [128,134,135]. The present minireview has illustrated the essential role of the micronutrient vitamin A for a specific but very critical early developmental event in building the embryonic heart. However, it is important to remember that the cardiovascular system is only one of many developmental events that require a regulatory input by vitamin A [128]. Finally, it is the overall nutritional experience and environment of the early embryo and fetus that determines the health status of the offspring [128,136]. 
